# Large Zenker’s Diverticulum: A Case Report

**DOI:** 10.7759/cureus.36783

**Published:** 2023-03-28

**Authors:** Shin T Zaw, Thinzar Zaw, Mahmudul Haque

**Affiliations:** 1 College of Medicine, Lake Erie College of Osteopathic Medicine, Bradenton, USA; 2 College of Medicine, University of Central Florida College of Medicine, Orlando, USA; 3 Gastroenterology and Hepatology, Lakeland Regional Health, Lakeland, USA

**Keywords:** diverticulotomy, esophagus, esophageal diverticulum, odynophagia, dysphagia

## Abstract

Zenker's diverticulum (ZD) is a type of esophageal diverticulum, a relatively rare disease in the pharyngoesophageal area. It is a pulsion diverticulum, or false diverticulum, located dorsally at the wall between the pharynx and esophagus. This area is known as Killian's triangle or dehiscence and is a region of relative weakness. Common symptoms of ZD include dysphagia, choking, persistent cough, loss of weight, hoarseness, halitosis, regurgitation of undigested food, and borborygmi within the cervical region. We are reporting a case of oropharyngeal dysphagia due to a ZD in a 65-year-old man with a history of worsening dysphagia for two years. Clinical presentation, diagnosis, and treatment options for ZD are discussed, along with the underlying pathophysiology of this condition.

## Introduction

ZD is a type of false diverticulum that develops at the wall between the pharynx and esophagus and is characterized by dysphagia as the most consistent symptom [1]. The cause of this acquired condition is thought to be a region of relative weakness in the wall of the pharynx and esophagus. This vulnerability is referred to as Killian's triangle or Killian's dehiscence, resulting in an outpouching of the mucosal and submucosal layers [2]. ZD is often classified by size, typically measured in the craniocaudal direction. The three size classifications are small (up to 2 cm), intermediate (2-4 cm), and large (greater than 4 cm) [3]. It is a relatively rare condition that predominantly affects men, with a prevalence of 0.01% to 0.11% in the general population [2]. The diagnosis is typically made during the seventh to eighth decades of life and seldom before age 40 [4].

The modified barium swallow, which uses contrast video fluoroscopy, is the most crucial imaging modality for diagnosing ZD [5]. Several surgical options are available for the treatment of ZD. Among these options, endoscopic diverticulotomy has been shown to be the most effective treatment due to decreased post-operative complications and mortality rates [6]. We present a case of a large ZD in an adult male, along with the diagnostic strategy and surgical treatment.

## Case presentation

A 65-year-old male patient presented to the clinic with a chief complaint of difficulty swallowing. The patient has been experiencing this problem for the last two years, and the symptoms have progressively worsened. The patient reported experiencing a choking sensation and regurgitation of undigested food and liquid into his mouth after eating and has to bend over to swallow. Additionally, the patient reported losing weight. There was no previous medical history, and the patient denied having diabetes.

On physical examination, the patient was well-developed and well-nourished. His neck was supple, and there was no adenopathy. The abdomen was soft, non-tender, non-distended, and without masses, and no organomegaly or hernias were present. Normal bowel sounds were noted.

**Figure 1:**
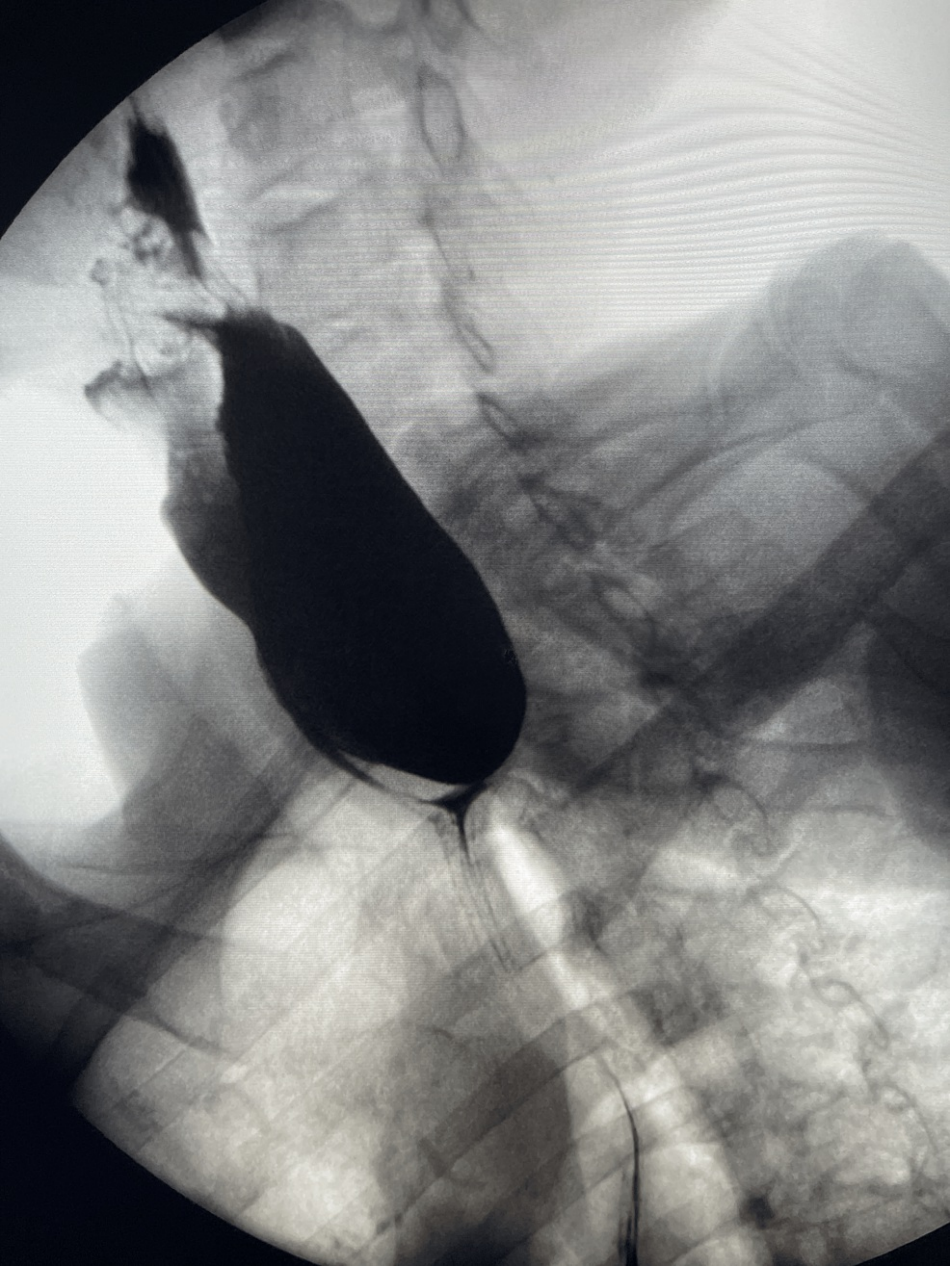
Preoperative barium swallow radiograph exhibits significant retention of barium

An EGD was performed, which revealed a single diverticulum with a large opening in the upper third of the esophagus, suggesting a diagnosis of ZD. The patient then underwent a barium swallow, which showed a large ZD in the cervical segment of the esophagus that was 7 cm long and contained undigested debris (Figure 1). The patient underwent endoscopic diverticulotomy with no complications and resolution of symptoms.

## Discussion

Observed by radiologic and endoscopic techniques, esophageal diverticula are relatively rare, with an incidence rate of 0.06% to 4% [7]. Esophageal diverticula may occur in the pharyngoesophageal area, mid-esophagus, or distally [7]. The three types of pharyngoesophageal diverticulums are ZD, Killian-Jamieson diverticulum (KJD), and Laimer's diverticulum (LD). The most prevalent type of diverticulum is ZD, accompanied by KJD and, lastly, LD [8]. ZD is a pulsion diverticulum, or false diverticulum, located dorsally at the wall between the pharynx and esophagus. This area is known as Killian's triangle or dehiscence. It is a region of relative weakness surrounded by the transverse fibers of the cricopharyngeal muscle and also the oblique inferior pharyngeal constrictor muscle [9]. It primarily affects men. Between 0.01% and 0.11% of the overall population is impacted by ZD [2]. Typically, the diagnosis is made during the seventh to eighth decades of life [4]. This patient presented in his mid-sixties.

ZD commonly presents with dysphagia, choking, persistent cough, loss of weight, hoarseness, halitosis, regurgitation of undigested food, and borborygmi within the cervical region [3]. The most common sign of ZD is trouble swallowing, also known as dysphagia, which can occur due to the failure of the upper esophageal sphincter to fully open or external compression of the cervical esophagus due to the presence of the diverticulum itself [1]. The patient experienced difficulty swallowing both solids and liquids, as well as regurgitating food that had not been properly digested. Boyce's sign, a rare physical examination finding suggestive of ZD, involves a swelling in the neck that gurgles on palpation [3]. However, upon physical examination, this patient had no palpable neck swelling.

The preoperative evaluation of a patient suspected of having an esophageal diverticulum typically involves a comprehensive workup which may include endoscopy, barium swallow, manometry, and pH monitoring [7]. The barium swallow is considered the most critical diagnostic tool of these tests. The modified barium swallow, also known as a videofluoroscopic swallow study, utilizes contrast video fluoroscopy with continuous monitoring of the swallowing mechanism and is more effective than the traditional single-shot barium swallow, which may overlook a small diverticulum [5]. Once ZD is diagnosed, a thorough endoscopic examination of the pouch is essential. This evaluation helps to exclude the presence of malignancy [10]. ZD is often classified by size, typically measured in the craniocaudal direction. The three size classifications are small (up to 2 cm), intermediate (2-4 cm), and large (greater than 4 cm) [3]. A normal barium swallow was performed in our case and demonstrated a large ZD (7 cm).

Various surgical procedures, including open diverticulectomy, endoscopic diverticulotomy, diverticulopexy, myotomy, and diverticular inversion alongside or without myotomy, have been described as treatments for ZD [11]. In the general population, the open surgical approach for treating ZD was associated with a higher incidence of postoperative complications, including but not limited to fistula development, hematoma, abscess, recurrent nerve paralysis, phonation difficulties, and Horner syndrome (10.5% versus 8.7%) [1,6]. Furthermore, it was also associated with a higher mortality rate (0.6% versus 0.2%) compared to the endoscopic approach. Endoscopic surgery also decreased the likelihood of permanent nerve damage, wound infection, prolonged hospitalization, and fistula development. However, intraoperative bleeding and esophageal mucosal injury increased [6]. Ultimately, the best approach will be determined case-by-case in consultation with the patient and the treating physician. The patient, in this case, was managed by endoscopic diverticulotomy.

## Conclusions

Esophageal diverticula are relatively rare, with ZD being the most common type. This patient presented with symptoms consistent with ZD, including dysphagia, odynophagia, and regurgitation of undigested food. The diagnosis was confirmed through a barium swallow, which revealed a large ZD. Treatment options include open surgical procedures and endoscopic diverticulotomy, with the latter being associated with lower rates of postoperative complications and mortality. Although ZD is not common in the general population, clinicians should consider it a possible cause when a patient complains of difficulty swallowing in the oropharyngeal area.
